# An Uncommon Case of Intraluminal Duodenal Diverticulum Complicated by Recurrent Gastrointestinal Bleeding and Small Bowel Obstruction

**DOI:** 10.7759/cureus.21391

**Published:** 2022-01-18

**Authors:** Hoang Ta, Dalbir Sandhu, Mohamad Mouchli

**Affiliations:** 1 Internal Medicine, Cleveland Clinic Akron General, Akron, USA; 2 Gastroenterology, Hepatology & Nutrition, Cleveland Clinic Main Campus, Cleveland, USA

**Keywords:** intraluminal duodenal diverticulum (idd), biliopancreatic manifestation, perforation, diverticulum, small bowel obstruction, by recurrent gastrointestinal bleeding, gastrointestinal bleed, duodenal diverticulum, windsock diverticulum

## Abstract

Intraluminal duodenal diverticulum (IDD) is a rare congenital anomaly resulting from the failure of the duodenum to recanalize during embryogenesis leaving a duodenal diaphragm or web within the lumen of the duodenum. In theory, the peristaltic force gradually stretches the tissue over time forming a diverticulum within the duodenal lumen. Identification of IDD by endoscopy or diagnostic imaging can be difficult, these lesions can be mistaken for other lesions or the collapsed diverticulum can be overlooked. The median age of presentation is the fourth decade. Although most cases are asymptomatic, some can present with vague abdominal complaints such as bloating, nausea, abdominal discomfort, or pain. Complications of IDD are intestinal bleeding, biliary pancreatic symptoms, intestinal obstruction, and perforation. Management of IDD complications could be challenging since data are limited. We present an unusual case of a 78-year-old female presenting with acute chest pain, palpitations, and incidental findings of IDD and pancreatic divisum. Her course was complicated by recurrent gastrointestinal bleeding and small bowel obstruction.

## Introduction

Intraluminal duodenal diverticulum (IDD) is a congenital anomaly caused by the failure of the recanalization of the duodenum during embryogenesis leaving a protruding diaphragm or web within the duodenal lumen. In theory, the resulting saccular protrusion or web can elongate over time from the mechanical traction exerted by peristalsis. As a result, the median age of presentation is the fourth decade of life [1]. Most of the IDD are found incidentally and rarely cause symptoms. IDD can present as vague gastrointestinal (GI) symptoms such as abdominal discomfort, bloating, early satiety, nausea, and or vomiting. Complications related to IDD are rare but may include hemorrhage, biliopancreatic manifestations, bowel obstruction, or perforation. This study provides a report of an incidental endoscopic finding of IDD and pancreatic divisum complicated by recurrent GI bleeding and small bowel obstruction. We did a basic literature review on how to manage each of these IDD complications reported on PubMed, Elsevier, and the American Journal of Gastroenterology database.

## Case presentation

A 78-year-old female with a history of diabetes mellitus, chronic obstructive pulmonary disease, peripheral vascular disease, hyperlipidemia, chronic back pain on opioids, and coronary artery disease, who presented to the emergency department with acute chest pain. She reported waking up abruptly by chest pain that radiated to her neck and right arm, accompanied by heaviness, palpitations, and diaphoresis. Her electrocardiogram revealed normal sinus rhythm and apparent chronic left bundle branch block without ischemic changes. Lab results were significant for elevated high sensitivity troponins 246, and B-type natriuretic peptide 3,544. Also, hemoglobin was 8.1, last known 11.1 (7 months prior to admission), hypokalemia 2.3, hypomagnesemia 1.4. Iron studies revealed iron deficiency anemia. She was admitted to the cardiovascular intensive care unit (CVICU), evaluated by cardiology who arranged for a left heart catheterization. She was started on a heparin drip along with acute coronary syndrome protocol and electrolytes repletion. However, later that evening repeat hemoglobin showed 6.9 from the initial 8.1. No overt signs of bleeding. Heparin was stopped. Gastroenterology was consulted.

**Figure 1:**
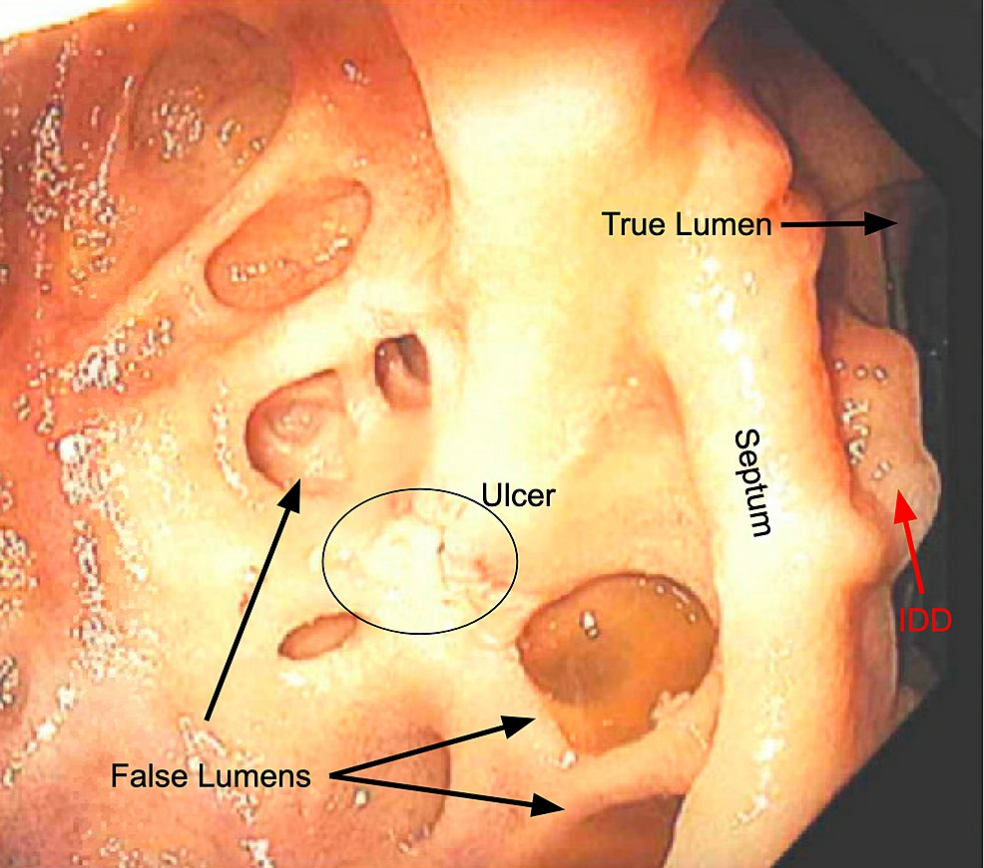
Upper endoscopy revealed triple lumens in the second portion of the duodenum. The first two lumens included numerous fenestrated diverticula. The third lumen contained an 8-cm clean-base ulcer (circled above) was treated with epinephrine injection (3/10,000), two hemoclips, and high-dose PPI. IDD can be visualized behind the septum. PPI: proton pump inhibitors; IDD: intraluminal duodenal diverticulum

In our initial investigation, she was relatively asymptomatic from a GI perspective. She denies abdominal pain, nausea, vomiting, melena, and hematochezia. No known history of GI bleeds or anemia (last known Hb was 11.1, 7 months before this admission). Her last colonoscopy was done 6 years ago and it was normal. No prior upper endoscopy was done. She is a current smoker with a 15-pack year history and denies non-steroidal anti-inflammatory drugs (NSAIDs) use. She underwent esophagogastroduodenoscopy (EGD), which revealed triple lumens in the second portion of the duodenum (Figure 1). The first two lumens included numerous fenestrated diverticula. The third lumen contained an 8-cm clean-base ulcer (Forrest 3) that was treated with epinephrine injection (3/10,000), two hemoclips, and high-dose proton pump inhibitors (PPI). An IDD is visualized behind the septum (Figure 1).

**Figure 2:**
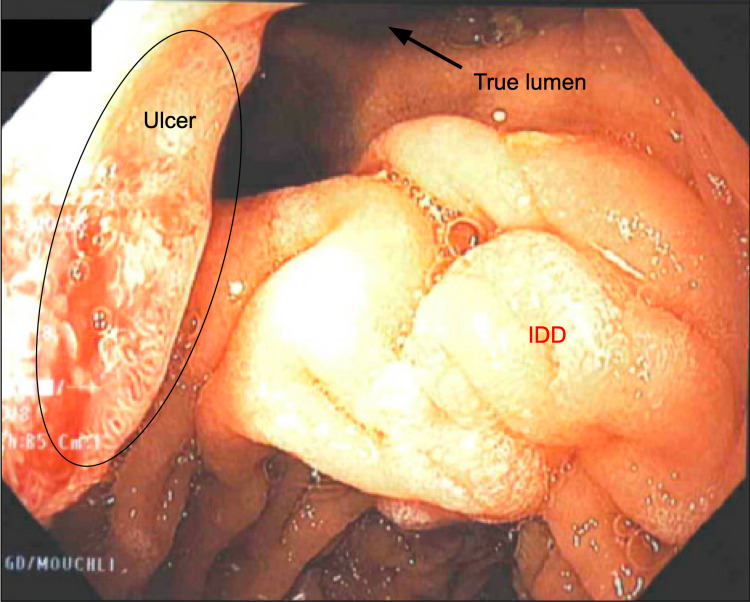
Esophagogastroduodenoscopy after 4 months. Revealed two large clean-based ulcers (8 cm, 5 cm) and multiple small ulcers and erosions in the duodenal bulb. This mass is a deflated IDD inverted on itself was partially dissected via needle-knife. IDD: intraluminal duodenal diverticulum

Four months later, she presented to the emergency department for complaints of epigastric pain. She endorsed poor appetite, continued weight loss, nausea, multiple episodes of non-bloody emesis, and constipation due to increasing dosage of chronic opiates for epigastric pain. She was admitted for concerns of upper GI bleed. EGD disclosed two large clean-based ulcers (8 cm, 5 cm), multiple small ulcers, and erosions in the duodenal bulb (Figure 2). Also, a deflated IDD inverted on itself was partially dissected into smaller pieces via a simple needle-knife. She was sent home on high-dose PPI twice daily.

**Figure 3:**
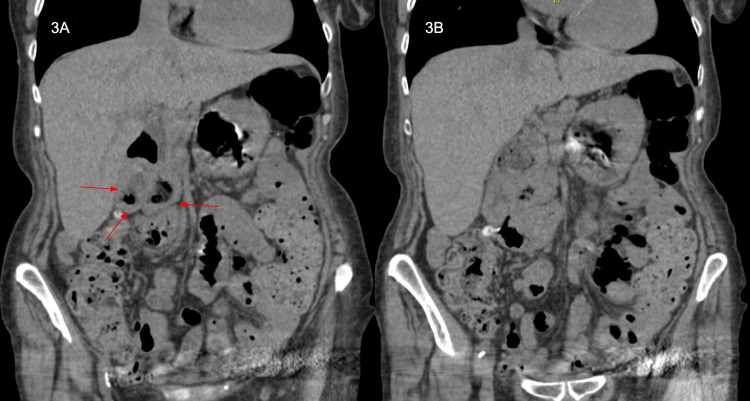
A-B: CT abdomen and pelvis without contrast in coronal view of intraluminal duodenal diverticulum (red arrows).

**Figure 4:**
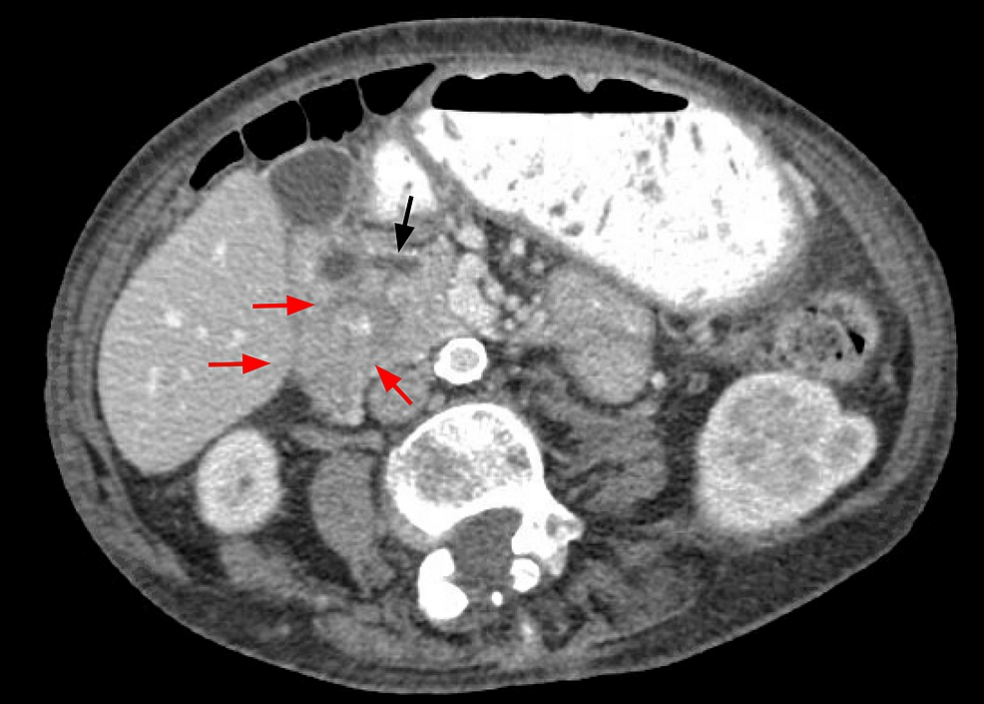
CT abdomen and pelvis with IV contrast in axial view. Shows the dilated pancreatic duct 5 mm (black arrow) and pancreas divisum emptying into a duodenal diverticulum. Intraluminal duodenal diverticulum (red arrows).

At 1-month follow-up, the patient continued to endorse abdominal pain with rebound tenderness and non-bloody emesis. A gastrin level was obtained, showing an elevated 367 (<115.0 pg/mL). We proceeded with a CT scan to rule out Zollinger-Ellison syndrome. The CT abdomen and pelvis disclosed a dilated common bile duct (CBD) and pancreatic duct (PD) with suspicion of pancreatic divisum (Figures 3-4). She underwent outpatient EGD, which revealed a large amount of undigested food in duodenal bulb outflow obstruction with significant D1/D2 stricture. In view of undigested food in the stomach and risk of aspiration, no dilation was done at that time. A few days later, we repeated EGD with Olympus gastroscope with controlled radial expansion (CRE) wire-guided balloon dilation catheter which could not be transverse through the D1/D2 tight angulation and stricture. A pediatric scope (XP190) was then exchanged which finally passed with great difficulty. A guidewire was then passed into descending duodenum but repeated attempts failed to dilate the CRE balloon due to the guidewire falling back repeatedly. In view of tight stricture, no improvement was seen with 10 mm dilation, further dilation was not attempted. An Olympus linear echoendoscope noted dilated CBD and PD with lobular pancreas with no obvious mass lesion or stone in CBD; although views were limited to D1/D2 stricture. The patient was offered surgical diverticulectomy, but she preferred conservative management.

## Discussion

IDD or “windsock” diverticulum is a rare congenital malformation arising from an incomplete recanalization of the foregut during the 4-8th week of embryogenesis resulting in a duodenal diaphragm or web [1-3]. Over time, peristaltic forces lead to progressive ballooning of the tissue forming a diverticulum within the lumen. For this reason, the median age of presentation is the fourth decade. Identifying IDDs by endoscopy or imaging can be difficult because these lesions can be mistaken for other lesions or the collapsed diverticulum can be overlooked. The majority of cases are asymptomatic; however, when symptoms arise, they tend to be vague complaints such as bloating, nausea, vomiting, and abdominal discomfort. Complications of IDD include intestinal hemorrhage, biliopancreatic manifestation, bowel obstruction, and perforation. We will review a basic literature review on how to manage each of these complications.

Hemorrhage

Our patient presented with multiple episodes of hemorrhage. She was found with iron deficiency anemia with normal blood urea nitrogen (BUN) and creatinine ratio suspecting a chronic slow bleed. Her initial acute onset blood loss is most likely secondary to heparin-induced. She was treated with epinephrine injection (3/10,000), two hemoclips, and high-dose Protonix. With the advances in endoscopy, various endoscopic interventions have been used for the treatment of bleeding duodenal diverticula, including epinephrine injections, hemoclips, bipolar coagulation, and argon plasma coagulation [1-6].

Biliopancreatic manifestation

IDD most commonly occurs near the ampulla of Vater; thus, it can manifest biliary and/or pancreatic complications. Our patient was found to have dilated CBD and PD on CT imaging due to the tight stricture from IDD. Endoscopic treatment is the preferable approach as a less invasive procedure without major adverse effects. Endoscopic techniques include simple needle-knife incision (diverticulotomy), partial snare resection (diverticulectomy), endoscopic retrograde cholangiopancreatography (ERCP), and sphincterotomy with balloon dilation [7-11]. Furthermore, a new endoscopic technique for diverticulectomy has been exploited with impressive results including a 2-channel endoscope [10] and an expandable metallic stent inserted into the diverticulum [11].

Obstruction

Due to gastric and duodenal obstruction in our patient, she was recommended surgical diverticulectomy, however, conservative management was requested per patient. Historically, surgical intervention was performed for gastric and bowel obstruction [1,12]. Nowadays, with advancements in endoscopy and imaging; endoscopic diverticulotomy or diverticulectomy is the treatment of choice. The endoscopy approach includes using needle-knife papillotomy, sphincterotomy, argon plasma coagulation, yttrium aluminum garnet (YAG) laser, and endostapler have been described [7-17]. In a study by Bhalla et al., the use of a novel technique reported as submucosal dissection scissors (SB knife, (Olympus, Shinjuku City, Tokyo, Japan)) and blended the longitudinal wall by electrocautery showed resolution of symptoms [14]. There are reported cases of conservative management in obstructive IDD with no clear definite results due to high recurrence of symptoms [14,18].

Perforation

A fearful complication of the untraversable duodenal diverticulum is bowel perforation. Duodenal perforation reported as often as 78% are seen within the second portion of the duodenum, within 2 cm from the ampulla of Vater [19,20]. Although, it is exceedingly rare with a total sum of 11 cases described in the literature as of 2012 [20]. The most common causes of perforation are diverticulitis 62%, followed by enterolithiasis (~10%) and ulceration [20]. Treatment is based on the patient’s clinical condition and hemodynamical stability whether pursuing conservative versus surgical interventions. Conservative treatment of perforated duodenal diverticula is an acceptable and safe strategy in stable patients without signs of peritonitis [20]. A step-up approach to percutaneous drainage or surgery can be applied if conservative management fails [19,20].

## Conclusions

IDD may be easily missed unless one particularly considers this entity in patients presenting with signs of abdominal complaints. IDD should be included in the consideration of rare causes of intestinal hemorrhage, biliopancreatic manifestation, bowel obstruction, and perforation. Prophylactic excision of asymptomatic IDD is not recommended. The preferred management for symptomatic complications is endoscopic diverticulotomy or diverticulectomy including needle-knife incision, argon plasma coagulation, YAG laser, partial snare resection, and many other techniques mentioned in our study. A step-up approach should be applied if conservative management fails.
